# Epidemiological profile of breast cancer patients: descriptive study at the Laâyoune Sakia El Hamra Region Oncology Center

**DOI:** 10.11604/pamj.2025.50.35.41885

**Published:** 2025-01-28

**Authors:** Darifa El Hairach, Sarah Drider, Imane Fadel, Hefdhallah Al-Aizari, Abdellatif Bour, Soad Khal-Layoun

**Affiliations:** 1 Laboratory of Biology and Health, Faculty of Sciences, Ibn Tofail University, Kenitra, Morocco,; 2 Laboratory of Natural Resources and Sustainable Development, Faculty of Sciences, Ibn Tofail University, Kenitra, Morocco,; 3 Laboratory of Plants, Animals Productions and Agro-industry, Faculty of Science, Ibn Tofail University, Kenitra, Morocco,; 4 Department of Chemistry, Faculty of Education, Dhamar University, Dhamar, Yemen

**Keywords:** Patients of Morocco, breast cancer, epidemiology, knowledge of grade

## Abstract

This research, conducted in the Laâyoune-Sakia El Hamra Region in southern Morocco from January 2021 to December 2022, aims to describe the epidemiological profile of cancer patients at the Laâyoune Oncology Center. By following 90 patients who were under observation at the oncology center, the study sought to provide comprehensive insights into their sociodemographic and clinical characteristics. The study used a two-part questionnaire administered to 90 cancer patients at an ocular oncology center. The first part collected sociodemographic data such as age, marital status and place of residence, and the second part focused on clinical details including stage of diagnosis and treatment. It was found that the incidence of cancer among women over the age of 50 years in this study was 54.4%. Grade 1 was classified as high-grade, while grades 2 and 3 were not negative for hormone receptors. No significant differences were observed between the two groups in terms of risk factors or clinical parameters. However, the placebo group showed a greater preference for mammograms. About 84.4% of patients were diagnosed at stage I, while 15% showed double, triple or squamous cell carcinoma. No significant time differences were observed. Treatment details were available for more than 90% of patients and 3.3% of them underwent surgery with chemotherapy and/or radiation. The study revealed that breast cancer among older women patients is more common than in younger women in the study area. The study confirms the need for specific strategies for cancer screening and management in this region and to take necessary measures.

## Introduction

In recent times, breast cancer has overtaken lung cancer as the most frequently diagnosed cancer worldwide, with around 2.3 million new cases identified in 2020. Despite this shift, breast cancer continues to rank among the top five causes of cancer-related deaths worldwide. Breast cancer is the most common non-cutaneous malignancy and the deadliest form of cancer among women in Morocco [1]. Breast cancer is known to be one of the tumors with the highest prevalence among other types of cancer, with rates ranging from 1.5% to 46% [2].

The psychological distress experienced by women with breast cancer is distinctive, as it is associated with both perceptions of cancer and its treatment and is often associated with pain and mortality. Furthermore, it is associated with the symbolism of the breast, which represents femininity, motherhood and sexuality [3,4]. These paired symbolic cues contribute to shaping the prevalence of depression within this susceptible population. However, underdiagnosis of this condition is a common occurrence among these patients, partly due to the perception that feelings of sadness and frustration are considered inherent risks associated with breast cancer in Morocco [5]. According to most researchers, breast cancer in very young women has distinct epidemiologic, diagnostic and prognostic characteristics. Genetic predispositions, in particular, as well as a less favorable local and general prognosis compared to postmenopausal women, have been documented in the literature, thus impacting the therapeutic approach [6]. At the national level, evaluation of treatment times is one of the concerns included in the first cancer plan launched in 2010. Action 54 of this plan aims to establish a system to monitor and evaluate patient care. However, in Morocco, detailed data on cancer treatment times are still lacking, especially after the implementation of this first plan [7,8]. The emotional turmoil experienced by women with breast cancer has a unique dimension. On the one hand, it is intricately connected to the perception of the disease and its treatment, frequently associated with pain and the awareness of mortality [9]. On the contrary, it is related to the symbolic importance of breasts, symbolizing femininity, maternity and sexuality. These dual symbolic references significantly contribute to shaping the potential for depression in this susceptible population [10]. However, this condition is commonly ignored in these patients, partially due to the perception that feelings of sadness and discouragement are considered commonplace and anticipated, when confronted with the reality of a cancer diagnosis [4,11]. The underdiagnosis of the specified condition, likely cancer, is attributed to the similarity of symptoms with depression. Symptoms such as weight loss, fatigue, and sleep disturbances are shared between these two conditions. This commonality creates a challenge in distinguishing between them, potentially leading to overlook cases. Underdiagnosis indicates that instances of the condition are not adequately identified. The overlap of symptoms contributes to this problem, as healthcare providers may misinterpret or overlook signs, especially when presented with symptoms commonly associated with both cancer and depression [12-14]. A comprehensive and nuanced approach is crucial in healthcare assessment to accurately differentiate and diagnose these conditions, ensuring appropriate and timely interventions.

The objective of this work was to identify the different epidemiological and clinical aspects of breast cancer in premenopausal women, in the Laâyoune Sakia El Hamra Region located in southern Morocco.

## Methods

### Study design

This study spans a period from January 2021 to the end of December 2022 and involves 90 patients who were followed at the Laâyoune Oncology Center. It was carried out in two departments of radiotherapy and oncology within the oncology hospital in the city of Laâyoune.

### Setting

**Figure 1:**
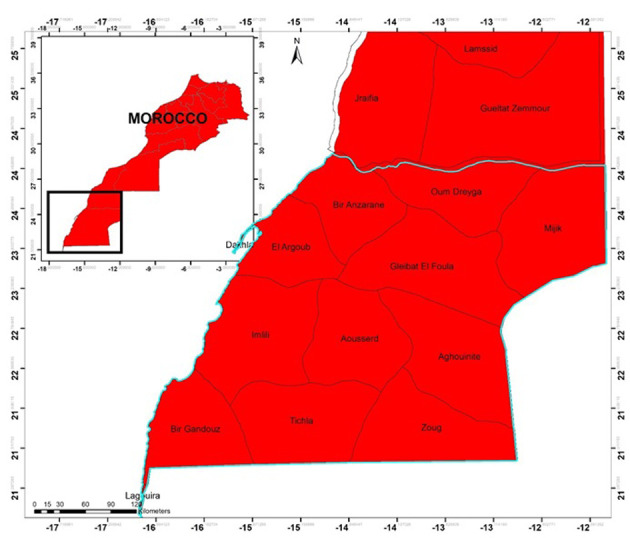
study area

The Laâyoune-Sakia El Hamra Region is primarily located in the western Sahara area in the southern part of Morocco and its administrative center is Laâyoune; covering an expanse of 140,018 square kilometers, it constitutes 19.7% of the national territory (Figure 1). From an administrative standpoint, the region comprises four provinces and 20 municipalities, consisting of five urban and 15 rural areas [15,16]. The population of the Laâyoune-Sakia El Hamra Region is 367,758 inhabitants, which is 1.1% of the total population of the country. In terms of employment sectors, the private sector holds the majority with 67.6% of the actively employed population, followed by the public sector, which represents 31.0% of this workforce. The study comprised 96 patients diagnosed with breast cancer in various stages. These participants were randomly selected and were briefed on the study objectives. Only individuals who provided voluntary and informed consent were considered for inclusion. Research involved women 18 and older who were well informed about their cancer, follow-up procedures and treatment. Exclusions included women who were less than 20 years old and those with poor general health. This study took place from January 2021 to the end of December 2022. Data were collected from 215 cancer patients at the cancer center in the capital of the Laâyoune-Sakia El Hamra Region. The study focused on 90 patients from the Eye Oncology Center.

### Variables

The data collected refers to the general characteristics (age, sex, marital status, medical insurance, region of origin) of the patients as well as clinical data (medical, surgical history, affected organs, etc.)

### Data resource

Cancer patient data was retrieved from the routine information system (ENOVA) implemented at the Laâyoune Oncology Center. Data collection involved a two-part questionnaire administered by a trained investigator. The initial section focused on sociodemographic characteristics such as age, marital status, residence and clinical details such as stage of diagnosis and administered treatment. The subsequent part included inquiries related to the Adaptive Coping Stra (AKU) scale.

### Data analysis

For statistical analysis, SPSS 21 software was utilized. Qualitative variable modalities were summarized in terms of absolute and relative frequencies.

### Ethical consideration

This retrospective study was approved by the regional health directorate and the Regional Oncology Center of Laâyoune Sakia El Hamra in Morocco, (version: January 22, 2021). In addition, the confidentiality of the information collected and the anonymity of the patients were guaranteed.

## Results

**Figure 2:**
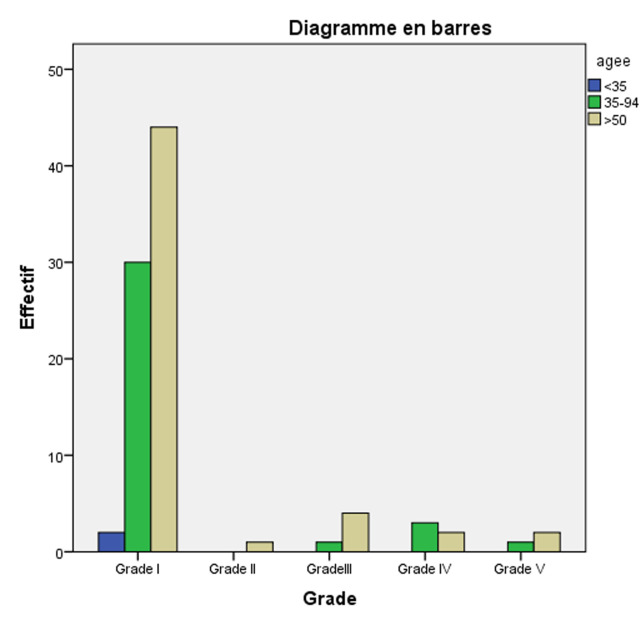
classification of breast cancer in the study area

A total of 90 patients diagnosed with breast cancer were treated at the Ophthalmic Oncology Center, almost all women and women over 50 years of age had the highest percentage of 58.9% (Table 1). 1 shows the distribution of the sample's cases according to marital status, showing that most of the sample are married at a rate of 64.4%, followed by single people at a rate of 10.0%, divorced people at a rate of 11.1%, and finally widows at a rate of 14.4%. There are 45 cases diagnosed with the primary source “CHIS”; which represents 50.0% of the total. Similarly, there is 1 case diagnosed through the screening program, representing 1.1% of the total. Finally, there are 44 cases diagnosed through the private sector, which makes up 48.9% of the total of persons. The different BMI categories in the table show the distribution of cases within these categories. The first row indicates that there is 1 case with a BMI less than 18.5, constituting 1.1% of the total. The second row shows that there are 27 cases in the BMI range of 18.5 to 24.9, making up 30.0% of the total, 29 cases in the BMI range of 25 to 29.9, making up 32.2% of the total, 32 cases in the BMI range of 30 to 34.9, making up 35.6% of the total, 1 case in the BMI range of 35 to39.9, making up 1.1% of the total (Table 1, Figure 2).

**Table 1:** sociodemographic characteristics

Sociodemographic characteristics	Sum	Percentage
**Age**		
<35	2	2.2
35-49	35	38.9
>50	53	58.9
**Marital status**		
Single	9	10
Divorce	10	11.1
Married	58	64.4
Widow	13	14.4
**Primary diagnosis (origin)**		
CHIS	45	50
Screening program	1	1.1
Private sector	44	48.9
BMI		
<18.5	1	1.1
18,5-24.9	27	30
25-29.9	29	32.2
30-34.9	32	35.6
35-39.9	1	1.1

Table 2 shows that most patients (82.2%) do not have a documented gynecological history, while a smaller percentage (17.8%) has a documented gynecological history. It also shows that most (82.2%) do not have a recorded surgical history, while a smaller percentage (17.8%) have a documented surgical history. It was also found that the vast majority (95.6%) did not have a documented personal record, while a small percentage (4.4%) had a personal record. Although it was revealed from the evidence provided by the patients that the vast majority (77.8%) had no recorded family medical history, a smaller percentage (22.2%) had a family medical history. As for Score Charlon, there are only five diseases of diabetes, one of which is hemoglobin, and Score Horse Charlon does not suffer from depression except one. Regarding current treatment, it turns out that most diseases are not treated immediately, at a rate of 81%, while the remaining 10% receive treatment, and the others receive other treatments, such as diabetes treatment. Through the results, it was found that 90% of the sample studied had no allergies, while 10% had skin sensitivities. Regarding stage and degree at diagnosis, 84.4% of cases were diagnosed at stage I and degree I. Medical oncology was administered to seventy-eight Hoyt patients (86.7%), while 3 patients (3.3%) underwent chemoradiotherapy, 3 patients (3.3%) received medical oncology and 6 patients (6.7%) underwent medical consultation.

**Table 2:** epidemiological characteristics of patients

Epidemiological characteristics	Sum	Percentage
**Gynaecological history**		
Non	74	82.2
yes	16	17.8
**Surgical history**		
Non	74	82.2
Yes	16	17.8
**Personal history**		
Non	86	95.6
Yes	4	4.4
**Family history**		
Non	70	77.8
Yes	20	22.2
**Score Charlon**		
None	83	92.2
Stroke	1	1.1
Diabetes	5	5.6
Hemiplegia	1	1.1
**Score Hors Charlon**		
None	88	97.8
Depression	1	1.1
Pleural tuberculosis	1	1.1
**Ongoing treatment**		
Diabetes	5	5.6
HTA	1	1.1
HTIC	1	1.1
Allergy		
Non	80	88.9
Yes	10	11.1
**Stade**		
Stade II	1	1.1
Stade III	5	5.6
Stade IV	5	5.6
Stade V	3	3.3
Stade I	76	84.4
**Grade**		
Grade I	76	84.4
Grade II	1	1.1
GradeIII	6	7.6
Grade IV	4	4.4
Grade V	3	3
**Service treatment**		
Radiochemotherapie	3	3.3
Medical oncology	78	86.7
Palliative care	6	6.7
Consultation	3	3.3

## Discussion

Breast cancer is a multifaceted disease, influenced by various factors, including genetic mutations (e.g. BRCA1, BRCA2), hormonal influences, age, and gender. Variations in the disease, such as different subtypes, guide treatment decisions. Environmental and lifestyle factors, such as diet and physical activity, contribute to population variation [2].

Geographic and ethnic differences, along with the complexity of the tumor microenvironment, further contribute to heterogeneity. Treatment responses vary and are influenced by factors such as molecular subtypes and biomarkers. The intricate interplay among these elements underscores the need for personalized approaches to prevention and treatment, as ongoing research seeks to unravel the complexities of breast cancer for improved outcomes. The expression of estrogen receptors and progesterone receptors, together with Her2 factor, varies. Identifying each tumor allows for the selection of appropriate treatment options, considering cost-effectiveness. In this study, all tumors were analyzed at the Histopathology Laboratory in Laâyoune, classified according to the SBR system. Most people with breast cancer are women over the age of 50 years, with a higher average age, and this is similar to other studies conducted in Casablanca, Rabat, and Fez [17-19]. The results showed that in this study, the cancerous tumor according to the SBR system is not very high compared to other studies. The upper part of the tumor is very high, according to what has been published in some research [17,20-22]. The results of our study align with findings from previous research conducted in other populations, which also reported an inverse relationship [17-19]. The impact of breast cancer poses a significant challenge for both developed and developing nations. However, citizens in developing countries face increased vulnerability due to delayed diagnosis and insufficient financial resources for adequate treatment, particularly affecting women with limited income.

According to data from the International Agency for Research on Cancer, breast cancer is the most prevalent cancer type in Morocco, accounting for 36.5% of all incidences and 19.7% of mortalities [23]. Despite these alarming statistics, the lack of accurate national registry records and data hinders the country from implementing an optimal strategy to minimize the burden of breast cancer, given the constraints of limited resources.

## Conclusion

Breast cancer among young Moroccan patients is increasing, which requires comprehensive epidemiological studies to understand this notable increase and investigate associated factors. Although its clinical presentation and progression may not differ significantly from that of older women, it often exhibits more aggressive biological characteristics, a higher genetic predisposition, and a delayed diagnosis. Breast cancer in the younger demographic can feature additional genetic and biological traits that make it more aggressive, raising additional challenges in the course of treatment. There is also a higher genetic predisposition to the disease within this age group. Delayed diagnosis proves to be a significant challenge, underscoring the importance of awareness and early detection. Late diagnosis could contribute to the advancement of disease in young people, affecting treatment options and final results. In general, the medical community and researchers need to shed light on this growing challenge, identifying the causative factors and biological impacts of breast cancer among young people. This is crucial to improve prevention and treatment strategies tailored to this vulnerable age group.

### What is known about this topic

- Breast cancer is more prevalent among older women than younger women in the study area;
- The results indicate that older women are at greater risk of breast cancer and that screening and focused management strategies are urgently needed to address this risk.

### What this study adds

- The study provides significant insights into the epidemiology of cancer in the Laâyoune-Sakia El Hamra Region, with a particular emphasis on the prevalence of breast cancer among older women, highlighting the necessity for dedicated research and healthcare strategies aimed at this group;
- It underscores the importance of understanding cancer's epidemiological characteristics in the region, focusing on early detection and advocating for specialized healthcare interventions tailored to older women.
